# Zika Virus Infection Alters Gene Expression and Poly-Adenylation Patterns in Placental Cells

**DOI:** 10.3390/pathogens11080936

**Published:** 2022-08-18

**Authors:** Stephanea L. Sotcheff, John Yun-Chung Chen, Nathan Elrod, Jun Cao, Elizabeth Jaworski, Mugé N. Kuyumcu-Martinez, Pei-Yong Shi, Andrew L. Routh

**Affiliations:** 1 Department of Biochemistry & Molecular Biology, University of Texas Medical Branch, Galveston, TX 77555, USA; 2 Beijing Key Laboratory of Environmental and Viral Oncology, Faculty of Environment and Life, Beijing University of Technology, Beijing 100124, China; 3 Department of Neuroscience, Cell Biology and Anatomy, University of Texas Medical Branch, Galveston, TX 77555, USA; 4 Sealy Institute for Drug Discovery, University of Texas Medical Branch, Galveston, TX 77555, USA; 5 Sealy Center for Structural Biology and Molecular Biophysics, University of Texas Medical Branch, Galveston, TX 77555, USA; 6 Institute for Human Infections and Immunity, University of Texas Medical Branch, Galveston, TX 77555, USA

**Keywords:** alternative polyadenylation, Poly(A)-ClickSeq, zika virus, placenta, microcephaly

## Abstract

Flaviviruses are small RNA viruses that are mainly transmitted via arthropod vectors and are found in tropic and sub-tropical regions. Most infections are asymptomatic (90–95%), but symptoms can be as severe as hemorrhagic fever and encephalitis. One recently emerged flavivirus is Zika virus (ZIKV), which was originally isolated from rhesus monkeys in Uganda roughly 70 years ago but has recently spread east, reaching S. America in 2015–2016. This outbreak was associated with the development of Guillain–Barré syndrome in adults and microcephaly in infants born to expectant mothers infected early in pregnancy. ZIKV must traverse the placenta to impact the development of the fetus, but the mechanisms responsible are unknown. While flaviviruses are known to disrupt splicing patterns in host cells, little is known about how flaviviruses such as ZIKV impact the alternative polyadenylation (APA) of host transcripts. This is important as APA is well-established as a mechanism in the regulation of mRNA metabolism and translation. Thus, we sought to characterize transcriptomic changes including APA in human placental (JEG3) cells in response to ZIKV infection using Poly(A)-ClickSeq (PAC-Seq). We used our differential Poly(A)-cluster (*DPAC*) analysis pipeline to characterize changes in differential gene expression, alternative poly-adenylation (APA) and the use of alternative terminal exons. We identified 98 upregulated genes and 28 downregulated genes. Pathway enrichment analysis indicated that many RNA processing and immune pathways were upregulated in ZIKV-infected JEG3 cells. We also updated *DPAC* to provide additional metrics of APA including the percentage-distal usage index (PDUI), which revealed that APA was extensive and the 3′ UTRs of 229 genes were lengthened while 269 were shortened. We further found that there were 214 upregulated and 59 downregulated poly(A)-clusters (PACs). We extracted the nucleotide sequences surrounding these PACs and found that the canonical signals for poly-adenylation (binding site for poly-A binding protein (PABP) upstream and a GU-rich region down-stream of the PAC) were only enriched in the downregulated PACs. These results indicate that ZIKV infection makes JEG3 cells more permissive to non-canonical poly-adenylation signals.

## 1. Introduction

Flaviviruses are arthropod-borne single-stranded positive-sense RNA (+ssRNA) viruses that plague regions harboring their respective tick and mosquito vectors. Of the roughly 70 species of flaviviruses discovered to date, about half of them are mosquito-borne, including dengue (DENV), yellow fever (YFV), West Nile (WNV), Japanese encephalitis (JEV), and Zika (ZIKV) viruses [1,2]. Although many infections are asymptomatic, symptoms can range from febrile illness to hemorrhagic fever, encephalitis, or even death. These viruses are an increasing public health risk with the geographic expansion of *Aedes* mosquitoes caused by global warming [3,4,5,6]. There exist few vaccines against flaviviruses, the most notable being the YFV vaccine (17D) [7]. Dengue infects roughly 400 million people worldwide each year, across four serotypes. With a vaccine that has only shown efficacy for two serotypes and the potential for enhanced illness in naïve children who are previously uninfected by the dengue virus, a need for improved and efficacious vaccine strategies remains [8,9]. Furthermore, at present, there are no FDA-approved antivirals against flaviviruses. It is clear that a greater understanding of the molecular mechanisms of flavivirus infection is necessary to combat and protect against these human pathogens.

**Figure 1:**
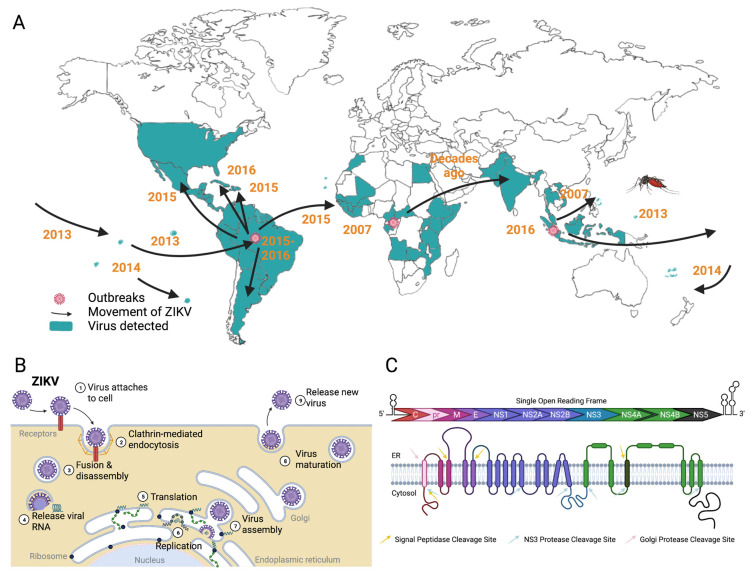
ZIKV global distribution over time and molecular biology. (**A**) Distribution of ZIKV infections and outbreaks over the last few decades, originating in Africa and spreading east towards SE Asia and the Americas. Note that geographic expansion of arthropod vector *Aedes* mosquitos plays an additional role. Adapted from Weaver mBio 2017 [10] with permission. (**B**) ZIKV replication cycle. (**C**) ZIKV genome and polyprotein.

In addition to being transmitted via mosquito bite, ZIKV can be transmitted both sexually and perinatally. ZIKV was originally isolated from rhesus monkeys in the Zika forest (Uganda) roughly 70 years ago. Over the last two decades, we have seen the eastern movement of ZIKV out of Africa, first to SE Asia, then to the Americas [10] (Figure 1A). Moreover, the 2015–2016 outbreak in South America was associated with an increased risk of development of Guillain–Barre syndrome in adults and microcephaly in infants born to mothers infected early in pregnancy [2,11]. This outbreak appeared to result in a strain that could persistently infect the testes, meaning that males can pass the virus on to their partners for months after infection. Although this outbreak incurred the largest number of ZIKV infections observed (~80 k cases) at a given time, it is important to consider the change in transmission route and consider how this might affect public health in the future. Recent studies have elucidated the mechanism of how ZIKV crosses the maternal–fetal interface [12,13,14,15]. These studies provide insight into flavivirus pathogenesis that may aid in the development of vaccines or antivirals in the future, not just against ZIKV but other flaviviruses as well.

ZIKV, similar to other flaviviruses, is a +ssRNA virus with an ~11 kb genome packaged into 50 nm enveloped particles. ZIKV enters the host cell via receptor-mediated endocytosis [1,2,16] (Figure 1B). Fusion with the endosomal membrane releases the viral nucleocapsid into the cytosol where uncoating occurs when the viral genome is freed from the capsid proteins. This genome contains a single open reading frame (ORF) that can be immediately translated at the endoplasmic reticulum (ER) membrane after uncoating, producing a viral polyprotein [11,17,18,19] (Figure 1C). The viral polyprotein is co- and post-translationally cleaved to generate ten viral proteins, three structural (capsid, membrane [prM], and envelope) and seven non-structural (including the viral RNA-dependent RNA polymerase [RdRP] and protease). At that point, the positive-sense genome can be translated or serve as a template to make negative-sense RNA. In any case, there are the following three soluble viral proteins in the cytoplasm: capsid (C), protease/helicase (NS3), and RdRP (NS5). Following synthesis of the nascent positive-sense genome in the cytoplasm, viral RNA is encapsidated at the ER membrane and immature particles begin budding in the ER lumen. These particles are exported from the cell via the secretory pathway through the Golgi, where furin cleavage of prM produces mature particles ready to be expelled from the cell via exocytosis.

ZIKV infection has been shown to alter the host transcriptome in various cell types, in both mammals and mosquitos [12,14,20,21,22,23,24]. The findings of many such studies suggest that lipid and ceramide metabolism and innate immune response are upregulated following infection, especially in cells such as HEK-293. Interestingly, for many cell types, it is also seen that pathways involved in development are perturbed. This is consistent with the fact that ZIKV infection is associated with microcephaly caused by perinatal infections. Thus, it is important to characterize infection of the placenta, which should serve as a barrier to infection for the fetus, as well as fetal neuronal tissue, which has impeded growth due to ZIKV infection. Previously, a study compared ZIKV-elicited gene expression patterns in human-induced pluripotent stem cells (hiPSCs) induced into trophoblasts from dizygotic twins discordant for congenital Zika syndrome (CZS), a group of birth defects that are associated with ZIKV infection during pregnancy [12]. Their findings suggested that although interferon gene expression was not differentially expressed in the CZS trophoblasts in response to ZIKV infection, there was a significant increase in interferon Lambda 1 (IFNL1) secretion from the non-affected twin, but with no increase observed in trophoblasts from the CZS twin. This indicated that the CZS twin’s trophoblasts had an impaired ability to migrate, recruit immune cells, and control the viral infection. To investigate the impact of ZIKV infection in neuronal cells, some groups have performed transcriptomics studies in SH-SY5Y (neuroblastoma) cells, human neural stem cells (hNSCs), or human neural progenitor cells (hNPs) [22,23]. RNA-seq has also been used to identify alternative splicing (AS) isoforms caused by ZIKV infection. One such study conducted in hNPs showed AS events were enriched in genes associated with cell death, RNA processing, transport, and neuron development [22]. It was shown that dengue virus NS5 associates with the spliceosome and alters splicing patterns, shedding light on a potential mechanism for alternative splicing upon ZIKV infection [25]. It was later shown that ZIKV and JEV NS5 proteins interact with the spliceosome as well [26]. ZIKV has also been shown to alter host splicing patterns [20,22], which may be a result of the NS5:spliceosome interaction.

Previous studies have investigated different aspects of ZIKV infection in human placental (JEG3) cells [27,28]. In this study, we investigated the impact of ZIKV infection on the transcriptome of human placental cells (JEG3) at 16 h post-infection (hpi). This time point is sufficient for the virus to begin inducing transcriptomic changes but not for these changes to result in cell death. This is important to understand how the infection of ZIKV may impact the fetus. To determine differentially expressed genes (DEGs) associated with ZIKV infection, we used Poly(A)-ClickSeq (PAC-Seq) to characterize alternative poly-adenylation (APA) patterns that may affect gene expression by altering stability and/or regulation of mRNA transcripts [29,30]. We updated our Differential Poly(A)-Cluster (*DPAC*) analysis pipelines [31] to calculate percent distal usage (PDU) for each terminal exon to investigate APA for each mRNA 3′ UTR as well as differential use of individual poly(A)-clusters (PACs). We found evidence for extensive APA upon ZIKV infection, including in numerous spliceosomal genes. We further found 214 upregulated and 59 downregulated PACs. Scrutinizing the nucleotide sequences surrounding these PACs revealed that the canonical signals for poly-adenylation (the AWUAAA binding site for PABP upstream and the GU-rich region down-stream of PACs) were only enriched in the downregulated PACs.

Additionally, our results suggest that additional interactions may be responsible for the changes in splice patterns than those previously reported for both DENV and ZIKV. In this ZIKV study, we show that regions surrounding upregulated PACs contain the motif GGAAGAA, a binding site for various splicing factors and HNRNPs, and the various spliceosomal genes are found to undergo APA.

## 2. Materials and Methods

### 2.1. Cell Cultures and Reagents

Rat myoblasts from embryonic heart tissue (H9c2) and human placental cells (JEG3) were used for this study. We obtained RNA samples from JEG3 cells for sequencing. JEG3 cells were maintained in Dulbecco’s modified Eagle’s medium (11965-092, Gibco) supplemented with non-essential amino acids (11140-050, Gibco), sodium pyruvate (58636, Sigma), 10% fetal bovine serum (FBS, ATCC 30-2020), 1% pen-strep (P/S, Thermofisher Scientific 15140122), and HEPES (15630-080, Gibco). H9c2 cells (ATCC CRL-1446) were used in siRNA knockdown experiments. They were maintained in DMEM, supplemented with 10% FBS and 100 units/mL P/S. Both cell types were cultured at 37 °C and 5% CO_2_.

### 2.2. H9C2 Cells and KD of CPSF6

H9c2 cells were seeded at 10^6^ cells per 100 mm dish and transfected with scrambled siRNA (Thermo Fisher Scientific, Waltham, MA, USA, AM4611) or *Cpsf6* siRNA (Thermo Fisher Scientific siRNA ID #152434) at 20 nM using Lipofectamine RNAiMAX (Thermo Fisher Scientific 13778150). Cells were harvested 72 h post-transfection for RNA or protein extraction.

### 2.3. Viruses and Infections

**Figure 2:**
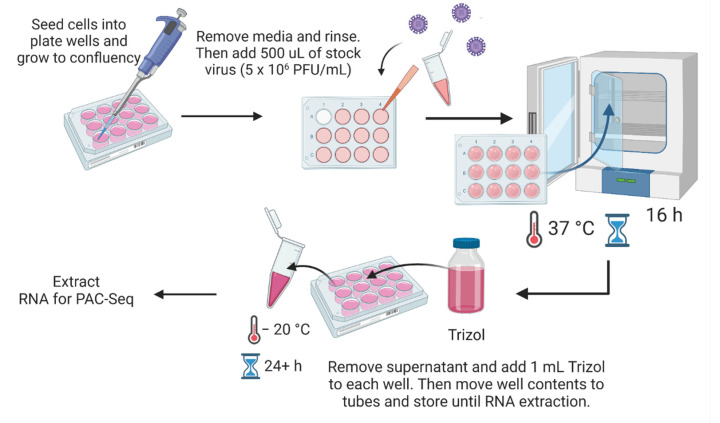
Infection and sample collection/storage prior to RNA extraction.

The ZIKV Puerto Rican strain (PRVABC59) was grown in African green monkey kidney (Vero) cells and stocks of virus were prepared by centrifuging the post-transfection media to remove cell debris, then aliquoting the supernatant. Titers of each passage were calculated by plaque assay. JEG3 cells were either mock-infected with media or infected with passage 2 (P2) PRVABC59 at a multiplicity of infection (MOI) of 3 plaque forming units (pfu)/cell in order to ensure the infection of all cells to obtain a representative RNA-seq profile of each condition (Figure 2). After adsorption of virus for 1 h at 37 °C, the virus-containing medium was removed and replaced with 2 mL fresh culture media. Infected cells were incubated at 37 °C and harvested at 16 h post-infection (hpi). The 16 hpi time point is sufficient for viruses to affect gene expression, but early enough to avoid a large percentage of cells undergoing apoptosis from skewing the transcriptomics results. Infections were confirmed by plaque assay in controls. Cell membranes were lysed and proteins degraded (to inactivate any remaining viruses within cells) by storing samples in TriZol reagent at −20 °C prior to RNA extraction.

### 2.4. RNA Extraction and Library Preparations

**Figure 3:**
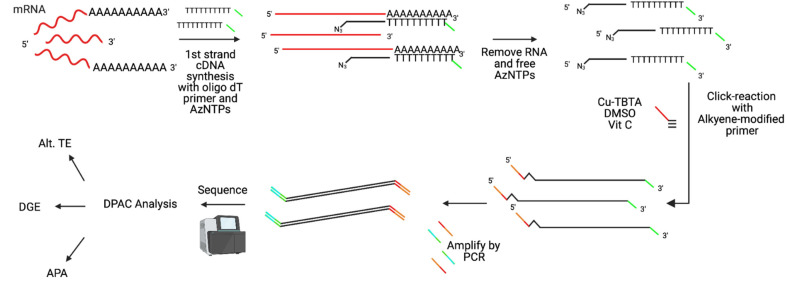
Preparation of PAC-Seq libraries from total cellular RNA (as described in [27,28]).

Total cellular RNA was isolated from thawed cells in TriZol using Direct-zol RNA Microprep kits (Zymo Research). RNA samples were quantified on a spectrophotometer (NanoDrop ND-1000; Thermo Scientific), with attention to the A260/A280 and A260/A230 ratios for quality. The short-read sequencing libraries were generated by Poly(A)-ClickSeq (PAC-Seq) [29,30] as outlined in Figure 3, starting with equal amounts of RNA for each library. This method uses an oligo(dT) primer to selectively reverse transcribe RNAs containing poly(A)-tails and includes azido-NTPs, which stochastically terminate RT, eliminating the need and biases for chemical or mechanical fragmentation. RNA integrity is less of a concern using PAC-Seq to prepare libraries from RNA compared to other RNA-sequencing methods as we are interested in just the 3′ end of mRNAs as opposed to full-length transcripts. A 5′-hexynyl-functionalized adaptor (IDT) is subsequently ‘click-ligated’ onto the 3′ ends of the azido-terminated cDNA fragments, yielding an ssDNA product flanked by the Illumina i5 and p7 adaptors. After a final PCR amplification step to add Illumina indexing barcodes, these libraries were gel purified (cut 200–400 bp) using a Zymo DNA Gel Extraction kit and then sequenced on an Illumina NextSeq550.

### 2.5. Differential Poly-A Cluster (DPAC) Analysis

*Differential Poly-A Cluster (DPAC)* version 1.21 was used to analyze the data from the short-read Illumina libraries [31]. DPAC can identify changes in overall expression, poly-adenylation or poly(A)-cluster (PAC) usage, and terminal exon usage. The overall pipeline has been previously described [31]. The command run for this data set was *DPAC-p PMCDB-t 4-x [flattened_annotations]-y [reference_names]-g [genome]-n 3-v human,hg19 [metadata_file] [index] [experiment name] [output_directory]*, where -*p* indicates parameters used, in this case P (perform data pre-processing), M (map data), C (force new poly-A cluster generation), D (perform differential APA analysis), and B (make individual bedgraphs), -t indicates how many threads were to be used, and -n indicates number of replicates. Before running D, it is necessary to generate a text file (metadata) that indicates which FASTQ files are associated with a particular treatment. For these studies we used annotations, gene names, and indices for rats or humans and mapped to the *Rattus norvegicus* (rn6) or *Homo sapiens* genome (hg19), as appropriate.

We updated the DPAC pipeline to provide additional metrics for the measurement of APA in PAC-seq datasets. Percentage Distal Usage (PDU) is calculated by measuring the ratios of the average normalized read count among each of the replicates from an experimental condition of the distal PAC to the proximal PAC of each exon, similar to other established approaches. For exons containing three or more PACs, the contribution of each PAC mapping in between the distal and proximal PACs is weighted according to its relative genetic distance from each PAC. So, where A%, B%, and C% are the relative abundances of the proximal, internal, and distal PACs, respectively, and x and x′ are the nucleotide distances between them, PDU is calculated by the following:

(1) $$\mathrm{PDU}:\;\left(0\;*\;A\%\right)+\left(\frac{x^{\prime }}{x+x^{\prime }}\;*B\%\right)+\left(1\;*\;C\%\right)$$

Change in PDU between two experimental conditions, called percentage distal usage index (PDUI), is simply the difference in the PDU measured for an exon between two experimental conditions. A negative PDUI denotes a net shortening of the 3′ UTR, while a positive PDUI denotes a net lengthening. If an exon is not sufficiently expressed (defined by –MinCounts) in any one of the replicates provided across the conditions, then PDU and PDUI are not calculated or reported for that exon.

To improve the usability of the *DPAC* pipeline, we have substantially overhauled the output file generated by *DPAC,* summarizing key information required by most users. Each gene occupies one row of a tab-separated (tsv) file. In the case that there are multiple exons with mapped PACs in a gene, each row is split using a Carriage Return Line Feed (CRLF) delimiter. We have found this to be compatible with most spreadsheet viewing software. However, in cases where CRLF is not recognized within a spreadsheet viewer or the data is exported to other graphing tools, we provide an alternative output where each exon is split into multiple rows. Finally, we implement *bedtools hyperlinks* to provide a convenient hyperlink for each annotated exon directly to the UCSC genome browser.

Tables highlighting which genes in with BioPlanet 2019 pathways are up or downregulated or have lengthened/shortened 3′ UTRs are included in Supplemental Materials (Data S3).

## 2.6. Data and Software Availability

Raw FASTQ datasets generated in this study are publicly available on the NCBI Small Read Archive with Project Numbers: PRJNA828166 (CPSF6 KD in H9c2) and PRJNA832287 (ZIKV in JEG3). Source code and example annotation datasets for the *DPAC* pipeline are freely available and maintained at https://sourceforge.net/projects/dpac-seq/ (accessed on 29 July 2022).

## 3. Results

### 3.1. Updates in Differential Poly(A)-Clustering (DPAC) Pipeline to Report Percent Distal Usage (PDU)

We previously developed the Differential Poly(A)-Clustering (*DPAC*) pipeline [31] to provide an all-in-one tool to preprocess and align 3′-end focused NGS data (such as from Poly(A)-ClickSeq). With these alignments, DPAC subsequently discovers and annotates poly(A)-clusters (PACs) in the 3′ UTR of mRNAs and uses *DESeq2* to determine whether PACs are significantly differentially expressed between two or more conditions. By calculating significant changes in PAC abundance, it is possible to simultaneously measure the change in gene expression and changes in alternative polyadenylation of an mRNA. Previously, *DPAC* provided a simple output reporting whether a gene exhibited APA with a ‘shortened’ or ‘lengthened’ 3′ UTR. APA was reported if one or more of the PACs in a 3′ UTR were determined to be differentially expressed between two conditions by DESeq2. If a 3′ UTR contained multiple PACs within a single 3′ UTR and an internal PAC was found to be differently expressed, this resulted in a more complex phenotype, denoted labeled as ‘both’. While this approach provides a strict statistical framework to determine whether there are significant changes in PAC usage, it does not quantify the relative usage of PACs within a terminal exon. Therefore, to bring *DPAC* in line with other tools that measure APA [32,33,34], we have substantially updated *DPAC* and overhauled the output report to use these same data to provide a robust metric of the relative degree of shortening or lengthening. Thus, the updated *DPAC* pipeline (as of version 1.10) provides a percent distal usage index (PDUI) for each mRNA terminal exon bearing one or more PACs, as described in Methods.

**Figure 4:**
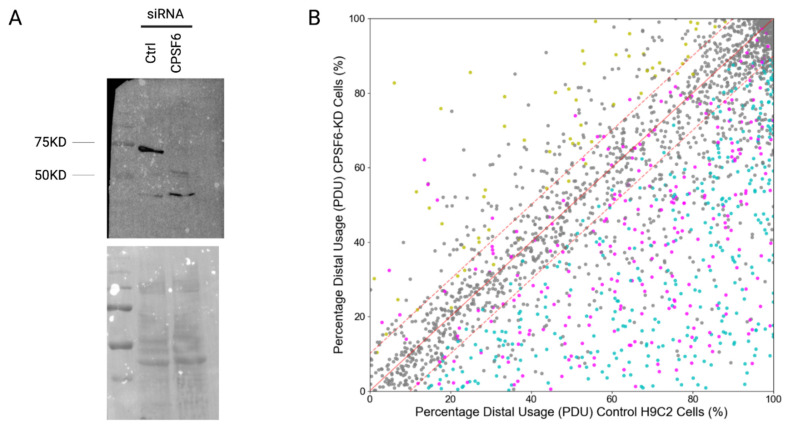
Validation of percent distal usage (PDU) calculations in *DPAC* by confirmed knockdown (KD) of CPSF6 in H9C2 cells. (**A**) Western blot confirming KD of CPSF6 (68 KD), which is present when the control siRNA is used, and absent when the siRNA targeting CPSF6 is used. (**B**) Percent distal usage plotted comparing control and CPSF6 KD cells, indicating that KD of CPSF6 results in overall shortening of 3′ UTRs. Each point corresponds to a one terminal exon, colored coded corresponding to whether the DPAC pipeline assigned APA (“*Shortened*”: blue; “*Lengthened*”: yellow; “*Both*”: magenta; no APA: grey).

To validate the updated *DPAC* pipeline, we knocked down (KD) a core component of the polyadenylation and cleavage machinery, CPSF6. CPSF6 binds to the upstream element (USE) near the poly(A) signal. KD of CPSF6 is well established to induce a 3′ UTR shortening phenotype [34,35]. CPSF6 siRNA KD was performed in rat heart myoblast (H9C2) cells, similar to our recent studies of APA in these cell types [36], and KD was confirmed by Western blot (Figure 4A). Total cellular RNA was extracted from both control and KD cells in culture, each in triplicate, using TriZOL and used as input for Poly(A)-ClickSeq library synthesis as previously described. We obtained >14 M raw reads per sample (Table S1), which were processed and mapped to the *rn6* genome by *DPAC* using default settings. We utilized the UCSC refSeq annotated mRNAs to create an annotated database of poly(A)-clusters (PACs) in the *rn6* genome (Data S1). By default, *DPAC* scrutinizes any PAC that contributes to at least 5% of all mapped mRNA 3′ ends of an exon in either experimental condition. APA is assigned if one or more of the PACs found within an exon exhibits a significant change (calculated by *DESeq2)* in abundance between the two conditions, as described in *Methods*. Upon knockdown of CPSF6, *DPAC* identified 740 exons with evidence of APA, dominated by shortening of 3′ UTRs (Figure 4, Data S1). The nature of the resulting APA changes for the 3′ UTRs that were called either “Shortened”, “Lengthened” or “Both” by DPAC was also compared to the PDUI calculation for every exon mapped in this dataset. PDUI is not calculated for an exon if the total read coverage over that exon in any one of the replicates is below 10 reads. The agreement in the assignment of APA with the calculation of PDUI is high, with no calls conflicting with one another (i.e., opposite calls), and with a small number of 3′ UTRs characterized as being “*Both*” reporting both negative (net shortening) and positive (net lengthening) PDUIs (Table 1, Figure 4). While these two contrasting methods to calculate APA thus broadly agree, each approach has its own advantages. The PDUI allows the ‘ranking’ of genes that exhibit the greatest extent of change in the overall 3′ UTR length, while the *DPAC* calls provide a strict assessment of APA based upon the statistically significant changes in individual PAC usage between conditions.

### 3.2. Differentially Expressed Genes in JEG3 Cells in Response to ZIKV Infection

**Figure 5:**
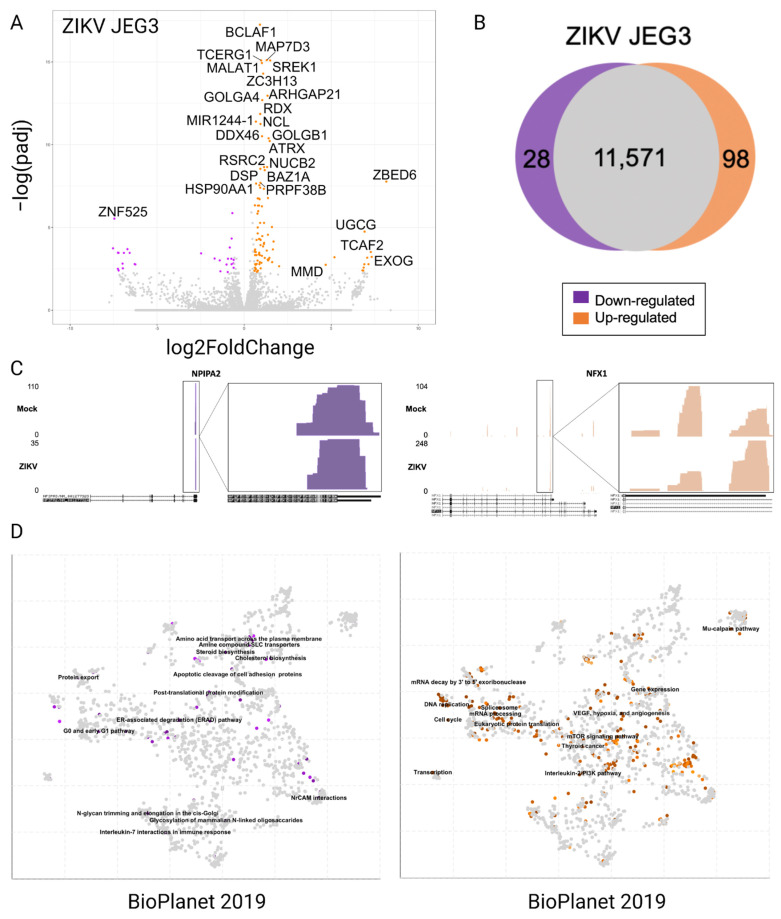
Differentially expressed genes upon ZIKV PRV infection of JEG3 cells. (**A**) Volcano plot for each time point, colored points indicating differentially expressed genes with a *p*-adjusted value < 0.1 and a log2FoldChange of greater than 0.58 (upregulated, orange) or less than 0.58 (downregulated, purple). (**B**) Overall count of differentially expressed genes with the previously mentioned statistics, compared to the total data set of 11,697 genes. As indicated by the legend, orange indicates upregulated, and purple indicates downregulated. (**C**) Bedgraphs of two genes that were differentially expressed: NPIPA2 (downregulated) and NFX1 (upregulated). Below each bedgraph is a map of the gene indicating introns/exons of alternative transcripts and the directionality of the gene. Note that PAC-Seq sequences from the poly-A tail or 3′ end of the gene. On the right we zoom in to find that there is a trailing off of the reads as they get further from the 3′ end, as expected. (**D**) Scatterplots from *Enrichr* indicating differentially expressed pathways or ontologies. (*Enrichr* is an online tool made available by the Maayan Lab at Mount Sinai). These results suggest that ZIKV infection results in the upregulation of mRNA decay, splicing and mRNA processing but downregulation of cell cycle control as well as the ERAD pathway and IL-7 interactions.

We infected plated human placental (JEG3) cells with ZIKV at an MOI of 3 to ensure most cells were infected. Cells were incubated with the virus for 16 h prior to RNA extraction. We used Poly(A)-ClickSeq (PAC-Seq) to generate 3′-end libraries of mRNAs from the total cellular RNA. The use of a targeted (oligo-dT) primer and azido-NTPs removed the need for a selection step prior to reverse transcription and fragmentation after cDNA synthesis, respectively. These libraries were sequenced using an Illumina NextSeq 550, yielding ~16 M reads per sample (Table S2). The data from these libraries were analyzed using our updated *DPAC* pipeline, which allows us to investigate differential gene expression as well as alternative polyadenylation (Data S2). Reads were quality filtered, poly(A)-tracts trimmed, and reads were mapped to the human genome (hg19) using HISAT2. Poly(A)-clusters (PACs) were discovered and annotated de novo and then enumerated in each dataset to generate a count table for genes, exons, and PACs. *DESeq2* analysis yielded 11,697 genes to compare mock to ZIKV-infected JEG3 cells. Of those, 126 genes were differentially expressed (28 down and 98 upregulated) with a *p*-adjusted value (*p*-adj) of <0.1 and an absolute value of log2 fold change (|log2FC|) greater than 0.585 (corresponding to a 1.5× fold change). The distributions of genes based on their *p*-adj and log2FC are shown in the volcano plot in Figure 5. We confirmed the data quality was sufficient by loading *bedgraph* files generated by the *DPAC* pipeline into the UCSC Genome Browser [37]. We expected that reads would (1) map to the 3′ ends of genes and (2) reads would trail off as they were further from the 3′ ends because different reads would be of varying lengths (a block would potentially indicate PCR duplication of a single strand/read). These criteria were met, as shown in Figure 5C for genes encoding NFX1 and NPIPA2, a nuclear transcription factor that binds x-box motifs and a nuclear pore complex interacting protein, respectively. *EnrichR* was used to identify the enrichment of different pathways, ontologies, or phenotypes in the 126 genes differentially expressed [38,39,40]. The results suggest that ZIKV infection cells hinder JEG3 cell cycle control, the ERAD pathway, and IL-7 interactions and promote mRNA decay and processing (as well as alternative splicing) and the IL-2 pathway.

To see if these features were observed in other related flaviviruses, we additionally performed a parallel study using Dengue Virus 2 (DENV) to infect JEG3 cells in culture under identical conditions and experimental approaches. *DESeq2* analysis yielded 10,724 genes, of those, 115 genes were differentially expressed (73 down and 42 upregulated) with a *p*-adj < 0.1 and a |log2FC| greater than 0.58, and the distributions of genes based on their *p*-adjusted and log2FC are shown in the volcano plots in Supplemental Figure S1. *Enrichr* was used to identify enrichment of different pathways, ontologies, or phenotypes in the 115 genes differentially expressed [38,39,40], suggesting that DENV infection hinders AKT signaling and loading of class I MHC peptides and promotes Toll-like receptor signaling and TGF-beta signaling in JEG3 cells.

### 3.3. Alternative Poly-Adenylation in ZIKV Infected JEG3 Cells

**Figure 6:**
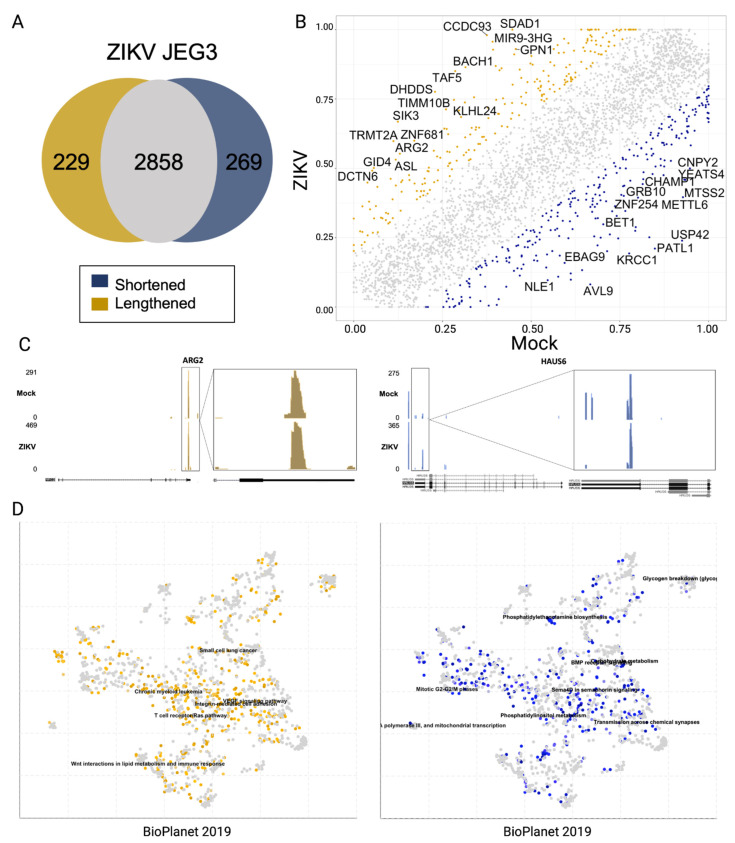
Alternative poly-adenylation (APA) in response to ZIKV infection in JEG3 cells. (**A**) Plots comparing the percent distal usage (PDU) of transcripts in the ZIKV or Mock condition for each time point. Blue–PDU decrease in ZIKV samples of 20% or more, indicating shortening of the 3′ UTR. Yellow–PDU increase in ZIKV samples of 25% or more, indicating lengthening of the 3′ UTR. Labelled transcripts have PDU changes greater than 50%. (**B**) Comparison of the number of identified poly-A clusters (PACs) per condition that were lengthened or shortened by 20% or more to the overall number of PACs. (**C**) Bedgraph files for ARG2 and HAUS6 indicating the mapping of PACs in these genes, which appear to be lengthened and shortened in response to ZIKV infection, respectively. (**D**) *Enrichr* scatterplots indicating enrichment of either shortening (blue) or lengthening (yellow) of 3′ UTRs of genes indicating that there appears to be shortening of transcripts involved in TGF-beta signaling and siRNA biogenesis and lengthening of transcripts involved in various aspects of the immune response as well as development and differentiation [38,39,40].

The same data from PAC-Seq libraries went through the remainder of the *DPAC* pipeline to allow investigation of APA as a result of ZIKV infection in placental cells [31]. *DPAC* identified 3356 genes with multiple PACs in the ZIKV-infected JEG3 cells (Figure 6A). For our analysis, we focused on changes of at least 20% in PDU and found 269 3′ UTRs shortened and 229 lengthened 3′ UTRs. To confirm, we uploaded the generated bedgraph files into the genome browser and viewed individual genes that were identified as having alternative poly-adenylation (APA), determined with our 20% PDU change cut-off [37]. Bedgraph files confirmed mapping to the human genome at the 3′ ends of genes and indicated that PTER (a gene producing a phosphotriesterase-related protein) and SURF4 (an integral membrane protein that interacts with ER-golgi intermediate compartment proteins) do indeed undergo shortening and lengthening of their 3′ UTRs, respectively (Figure 6C).

Generally, shortening of the 3′ UTR results in the removal of functional RNA motifs in the mRNAs, which can lead to altered regulation of the mRNA’s translation, stability, and sub-cellular localization, amongst other properties [41]. We used *EnrichR* to scrutinize lists of genes with terminal exons exhibiting PDU changes of greater than +/− 20%. As evidenced by the *EnrichR* Scatterplot Appyter plots (where each point is a gene set or pathway, and colored points are gene sets or pathways enriched with a *p*-value < 0.1) in Figure 6D (blue), there is a shortening of transcripts involved in siRNA biogenesis [38,39,40] and in post-transcriptional modifications of mRNA. In addition, these scatterplots show lengthening (yellow) of transcripts involved in differentiation, hedgehog and Wnt signaling, and B cell receptor signaling. This is interesting as these pathways are important for the development of a fetus, a concern here as CZS can cause a number of birth defects. Hedgehog signaling, for example, is responsible for patterning and cell fate determination in the central nervous system [42,43]. It is also responsible for tissue repair, which is important concerning damage caused by a viral infection of neuroprogenitor cells.

DPAC identified 2013 PACs in DENV infected JEG3 cells (Supplemental Figure S2) including 258 APA events (change in PDU > 20%). Our analysis found 132 3′ UTRs shortened and 126 lengthened 3′ UTRs. *EnrichR* indicated that transcripts with shortened 3′ UTRs were enriched in pathways involved in NOTCH signaling and actin cytoskeleton regulation [38,39,40]. In addition, transcripts with lengthened 3′ UTRs are enriched in pathways involved in protein processing in the endoplasmic reticulum (ER) and regulation of Toll-like receptor signaling.

**Figure 7:**
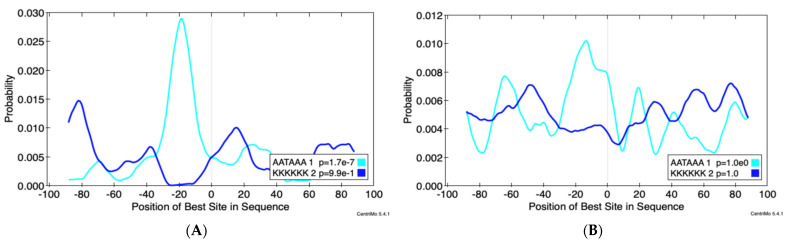
The ZIKV downregulated poly(A)-clusters (PACs) appear to use canonical poly-A signals, whereas the ZIKV upregulated PACs do not. (**A**) Centrimo (Bailey and Machanick, *NAR*, 2012) shows enrichment of AAUAAA within 100 nucleotides of the downregulated PACs; about 20 nucleotides upstream of the PAC. (**B**) There is slight enrichment of KKKKKK (a GU-rich region) downstream of the PAC. Neither of these sequences are enriched near the upregulated PACs. These results suggest that ZIKV changes the pattern of poly-adenylation on a global scale.

Additionally, we wanted to investigate if any RNA-binding protein motifs were present in proximity to the differentially expressed PACs in JEG3 cells upon ZIKV infection. To do this, we first extracted the sequences at various distances (100, 250, 500, 1000, and 2000 nts) surrounding PACs that were upregulated (i.e., preferentially used) and downregulated (i.e., relatively depleted) upon ZIKV infection in JEG3 cells. We analyzed these sequences with Discriminative Relative Expression Motif Elicitation (*DREME*) [44] to identify enriched motifs associated with either the up or downregulated PACs. Further, these motifs were cross-referenced with ‘the database of RNA binding specificities’ (RNA-binding protein database-RBPDB) [45] to identify RNA binding proteins that may interact near up or downregulated PACs. Within 100 nt of the upregulated PACs, we found the motif GGAAGAA, which is part of the motif for various splicing factors and HNRNPs, but we did not find enrichment of the canonical motif for poly-A binding protein 1 (PABP1, motif AAUAAA). This motif, however, was found enriched in the sequences between 100 and 250 nt of the downregulated PACs, about ~20 nt upstream of the PAC as expected (Figure 7A). We visualized the location of enrichment motifs relative to PACs using *CentriMo* [46]. In the plot, we also see some enrichment, although not to the same extent, of the KKKKKK motif (a GU-rich region) just downstream of the downregulated PACs. This indicates that the downregulated PACs are using canonical poly-adenylation signals. Neither of these sequences is enriched in the region surrounding the upregulated PASs (Figure 7B), suggesting an alternative mode for poly-adenylation.

We found a motif for PABPC1 (RAAGRAAA) enriched when we extended the distance to 250 nt of the upregulated PACs. As we extended the sequences from 500 to 2000 nt away from the upregulated PACs, we retained enrichment of this motif. We also found additional motifs that serve as binding sites for HNRNPL and SRSF3 (CACCACCA) and SRSF1/7 (AGCRAGAC). We found the motif GCCACCRC enriched between 1000 and 2000 nts from the downregulated PACs. This serves as a binding site for U2AF2 (GCCACCAC) as well as PABPC1 (GCCACCGC). These results suggest that the regions near upregulated PACs are more likely to interact with splicing factors than those that are downregulated.

## 4. Discussion

Although ZIKV was discovered in rhesus monkeys in the Zika forest of Uganda in the 1940s, it gained international attention during its emergence in Brazil in 2015–2016 [2,10,11]. This outbreak was associated with the development of Guillain–Barre syndrome in adults and microcephaly in infants born to women infected during pregnancy. Since then, it has been found that ZIKV can persistently infect the testes [47,48]. This is especially interesting as flaviviruses are typically transmitted via an arthropod vector, in this case, a mosquito bite. Now that we are aware that ZIKV can be transmitted both sexually and from mother to child during pregnancy, it is important to understand how these alternative transmission routes are made possible. For the virus to reach a fetus’ developing brain, it must first cross the placental barrier. Therefore, we have decided to investigate transcriptomic changes in ZIKV-infected human placental cells. It is also important to note that microcephaly does not occur upon infection of expectant mothers with other flaviviruses.

Our study used short-read sequencing to identify 126 DEGs and 498 APA genes in JEG3 cells at 16 hpi with ZIKV PRVABC59. We used *EnrichR* to determine if there was enrichment for any pathways or processes in these changes in the transcriptome [38,39,40]. This analysis identified an enrichment in ZIKV-induced upregulation of genes associated with interleukin and interferon signaling as well as metabolism and regulatory RNA pathways. In addition, there was enrichment of genes associated with cell cycle regulation and DNA replication and repair. Interestingly, we found that the 3′ UTRs of transcripts associated with the nuclear mRNA decay and splicing were shortened, whereas the 3′ UTRs of transcripts associated with various aspects of development and cell differentiation were lengthened.

The results from the DEG analyses are consistent with what has been previously noted by others, with the exception of the absence of upregulation of inflammatory pathways. However, this may be explained by the fact that 16 hpi is relatively early in infection. As noted above, there are a plethora of studies that have investigated changes in the host transcriptome upon ZIKV infection, focusing on changes in gene expression and splice isoforms. These studies have spanned across various cell types and have quite a few results in common. Notably, the upregulation of immune response and inflammation as well as lipid metabolism and the downregulation of genes involved in fetal development [12,14,20,22,23,24]. Although it is promising that these results seem to be rather conserved across various cell types, it also does not necessarily explain how infection of the placenta may be different from neural tissue or even tissues less concerning to human health with regard to ZIKV infection. One notable difference is the 3′ UTR lengthening of cell adhesion genes when infected with ZIKV, seen in our PAC-Seq results. Although interesting, as the development of microcephaly in infants born to ZIKV-infected mothers has been associated with dysregulation of inflammation, this suggests that the dysregulation of cell adhesion via integrins may have some impact on ZIKV infection.

A unique feature of this investigation is the use of Poly(A)-ClickSeq to characterize APA in response to flavivirus infection. We validated updates in *DPAC* calculating PDU and differential use of poly(A)-clusters (PACs) with analysis of PAC-Seq data generated from KD of CPSF6 (leading to overall 3′ UTR shortening). In the ZIKV JEG3 data, we found enrichment of genes associated with splicing and mRNA processing in transcripts with shortened 3′ UTRs, suggesting that these mRNAs are likely to be dysregulated. It has been previously noted that subgenomic flavivirus RNA (sfRNA), or even the NS5 protein interacting with the spliceosome, causes changes in splice patterns in response to flavivirus infection [49,50,51,52]. Our findings suggest that in addition, RNA degradation and miRNA biosynthesis appear to also be dysregulated, suggesting that ZIKV may be finding a way to either (a) evade these processes or (b) cause them to act on host transcripts and allow the translation of more viral proteins. Lengthened 3′ UTRs of transcripts associated with host immunity also suggest that ZIKV infection alters these UTRs to increase their potential for regulation, serving as an additional means of evading this response. Finally, the lengthening of transcripts important for fetal development and cell differentiation suggests that although these pathways may not be implicated in our DEG results, their altered 3′ UTRs potentially suggest their downregulation via increased regulation. Interestingly, we compared the genes that are differentially expressed and alternatively poly-adenylated and found little to no (maximum 4 out of 100) overlap between upregulation and either lengthening or shortening or downregulation and either lengthening or shortening.

Our findings suggest that changes in poly-adenylation may prove an important means of transcriptomic change in response to ZIKV infection. The APA results also suggest that ZIKV may alter the poly-adenylation patterns by shifting to a non-canonical signal for poly-adenylation. In the future, it will be interesting to identify how ZIKV induces these changes in APA, for example, via viral protein interactions with poly(A) polymerase or other factors involved in the poly-adenylation process. Alternatively, disruption of RNA-degradation by viral cofactors may also result in the observation of changes in transcript abundance measured in this study. Presumably, this would be one of the three soluble viral proteins, either NS5, NS3, or capsid. It may also be interesting to see how any of these soluble proteins may result in other changes by determining what nucleic acids they bind and any host proteins they interact with. Further studies with additional cell lines may elucidate how these changes in poly-adenylation patterns may lead to different disease phenotypes.

## Figures and Tables

**Table 1:** Comparison of exons called by *DPAC* to exhibit APA versus calculation of PDUI.

	−1 < PDUI < −0.1	−1 < PDUI < −0.1	−1 < PDUI < −0.1	PDUI Not Calculated
“Shortened”	327	31	0	43
“Both”	184	62	11	12
“Lengthened”	0	11	50	19
“No Change”	436	1289	192	8958

## Data Availability

All raw FASTQ data associated with this manuscript can be found on NCBI SRA under Project Number PRJNA828166 (CPSF6 KD in H9c2) and PRJNA832287 (ZIKV in JEG3).

## Supplementary Materials

The following supporting information can be downloaded at: https://www.mdpi.com/article/10.3390/pathogens11080936/s1, Figure S1: Differentially expressed genes upon DENV2 infection of JEG3 cells. (A) Overall count of differentially expressed genes with the previously mentioned statistics, compared to the total data set of 10,724 genes. As indicated by the legend, orange indicates up-regulated and purple indicates down-regulated. (B) Volcano plot highlighting differentially expressed genes (DEGs) in human placental (JEG3) cells in response to DENV infection. Each point is a gene. Up- (orange) and down-regulated (purple) genes have a *p*-adjusted value <0.1 and log2FoldChange of >0.585 or <−0.585 respectively. DEGs are labelled as space allows. (C) Bedgraphs of two genes that were differentially expressed: DDX5 (down-regulated) and DLG5 (up-regulated). Below each bedgraph is a map of the gene indicating introns/exons of alternative transcripts and the directionality of the gene. Note that PAC-Seq sequences from the poly-A tail or 3′ end of the gene. (D) Scatterplots from *EnrichR* indicating differentially expressed pathways or ontologies. (*EnrichR* is an online tool made available by the Maayan Lab at Mount Sinai). These results suggest that ZIKV infection results in the up-regulation of Toll-like receptor signaling and TGF-beta signaling but down-regulation of AKT signaling and loading of class I MHC peptides. Figure S2. Alternative poly-adenylation (APA) in response to DENV infection in JEG3 cells. (A) Comparison of the number of identified poly-A clusters (PACs) per condition that were lengthened or shortened by 20% or more to the overall number of PACs. (B) Plots comparing the percent distal usage (PDU) of transcripts in the ZIKV or Mock condition for each time point. Blue–PDU decrease in ZIKV samples of 20% or more, indicating shortening of the 3′ UTR. Yellow–PDU increase in ZIKV samples of 25% or more, indicating lengthening of the 3′ UTR. Labelled transcripts have PDU changes greater than 50%. (C) Bedgraph files for RBMS1 and NBR1 indicating the mapping of PACs in these genes which appear to be lengthened and shortened in response to ZIKV infection respectively. (D) *EnrichR* scatterplots indicating enrichment of either shortening (blue) or lengthening (yellow) of 3′ UTRs of genes indicating that there appears to be shortening of transcripts involved in NOTCH signaling and lengthening of transcripts involved in protein processing in the ER and regulation of Toll-like receptor signaling [38,39,40]; Table S1: Reads per sample for the H9c2 cells that were either mock- or CPSF6 siRNA treated. Table S2. Reads per sample for the JEG3 cells that were either mock- or ZIKV-infected. Data S1: Output of DPAC pipeline reporting APA and reads counts after KD of CPSF6 in H9c2 cells. Data S2: Output of DPAC pipeline reporting APA and reads counts after ZIKV infection of JEG3 cells (16 hpi). Data S3: Tables highlighting which genes in with BioPlanet 2019 pathways are up or downregulated or have lengthened/shortened 3′ UTR.
