# Supplementary material for: Zika Virus Infection Alters Gene Expression and Poly-Adenylation Patterns in Placental Cells
Source: Pathogens. 2022 Aug 18;11(8):936. doi: 10.3390/pathogens11080936 (PMC9414685; doi:10.3390/pathogens11080936)
Supplement: Supplementary file 1 [file pathogens-11-00936-s001.zip › pathogens-1734489-supplementary-tables & figures.pdf]

Table S1. Reads per sample for the H9c2 cells that were either mock- or CPSF6 siRNA treated.

Replicate	Cell Type	siRNA	Total Reads
1	H9c2	Ctrl	16′016′055
2	H9c2	Ctrl	14′144′524
3	H9c2	Ctrl	24′283′646
1	H9c2	CPSF6_KD	15′060′026
2	H9c2	CPSF6_KD	16′088′886
3	H9c2	CPSF6_KD	16′741′666

Table S2. Reads per sample for the JEG3 cells that were either mock- or ZIKV-infected.

Replicate	Cell Type	Virus	Time (hr)	Infected Y/N	Total Reads
1	JEG3	ZIKV PRVABC59	16	N	18,762,126
2	JEG3	ZIKV PRVABC59	16	N	16,704,921
3	JEG3	ZIKV PRVABC59	16	N	15,108,156
1	JEG3	ZIKV PRVABC59	16	Y	12,888,212
2	JEG3	ZIKV PRVABC59	16	Y	16,086,782
3	JEG3	ZIKV PRVABC59	16	Y	15,593,261

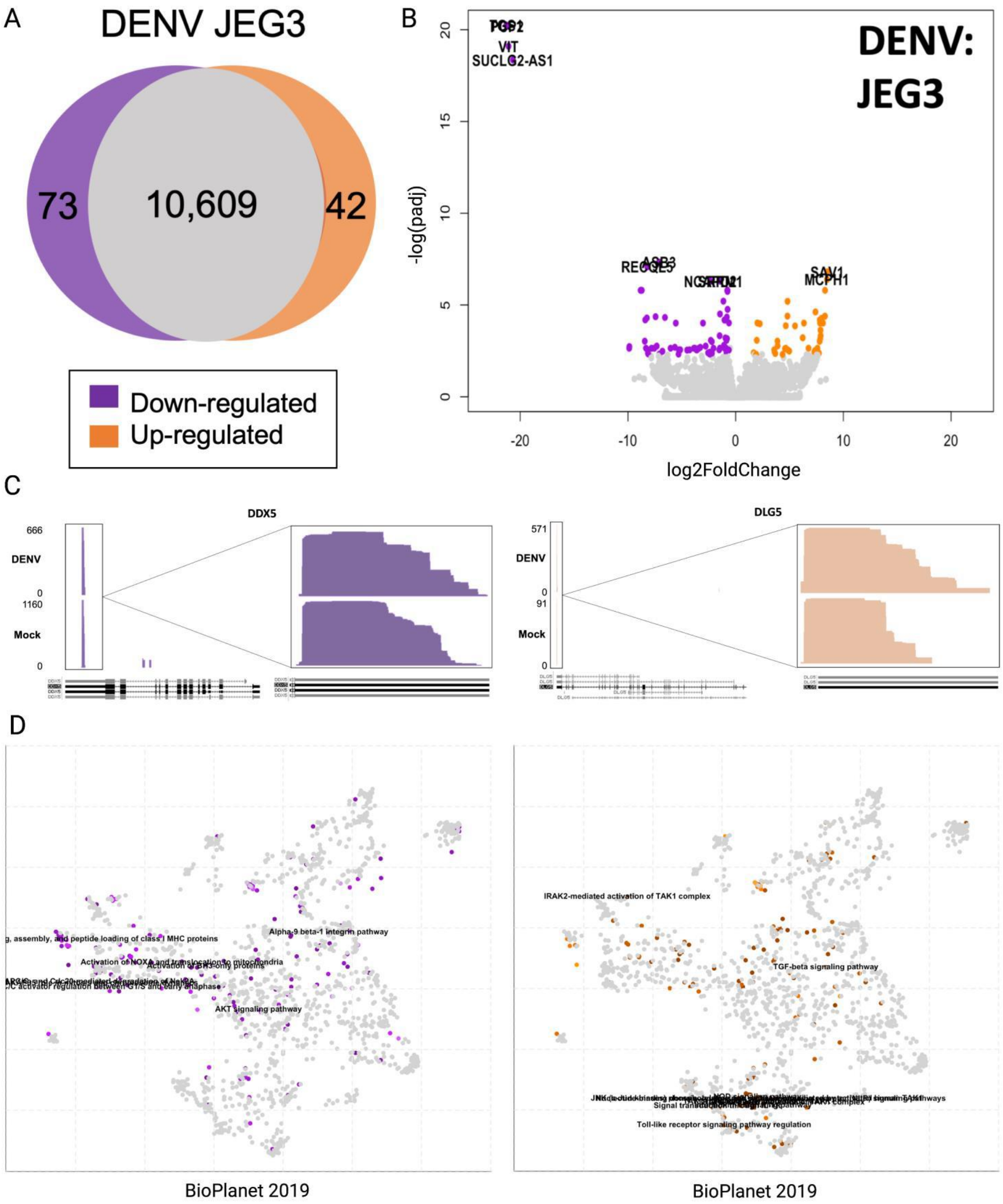

Figure S1. Differentially expressed genes upon DENV2 infection of JEG3 cells. (A) Overall count of differentially expressed genes with the previously mentioned statistics, compared to the total data set of 10,724 genes. As indicated by the legend, orange indicates up-regulated and purple indicates down-regulated. (B) Volcano plot highlighting differentially expressed genes (DEGs) in human placental (JEG3) cells in response to DENV infection. Each point is a gene. Up- (orange) and down-regulated (purple) genes have a *p*-adjusted value <0.1 and log2FoldChange of >0.585 or <−0.585 respectively. DEGs are labelled as space allows. (C) Bedgraphs of two genes that were differentially expressed: DDX5 (down-regulated) and DLG5 (up-regulated). Below each bedgraph is a map of the gene indicating introns/exons of alternative transcripts and the directionality of the gene. Note that PAC-Seq sequences from the poly-A tail or 3′ end of the gene. (D) Scatterplots from *Enrichr* indicating differentially expressed pathways or ontologies. (*Enrichr* is an online tool made available by the Maayan Lab at Mount Sinai). These results suggest that ZIKV infection results in the up-regulation of Toll-like receptor signaling and TGF-beta signaling but down-regulation of AKT signaling and loading of class I MHC peptides.

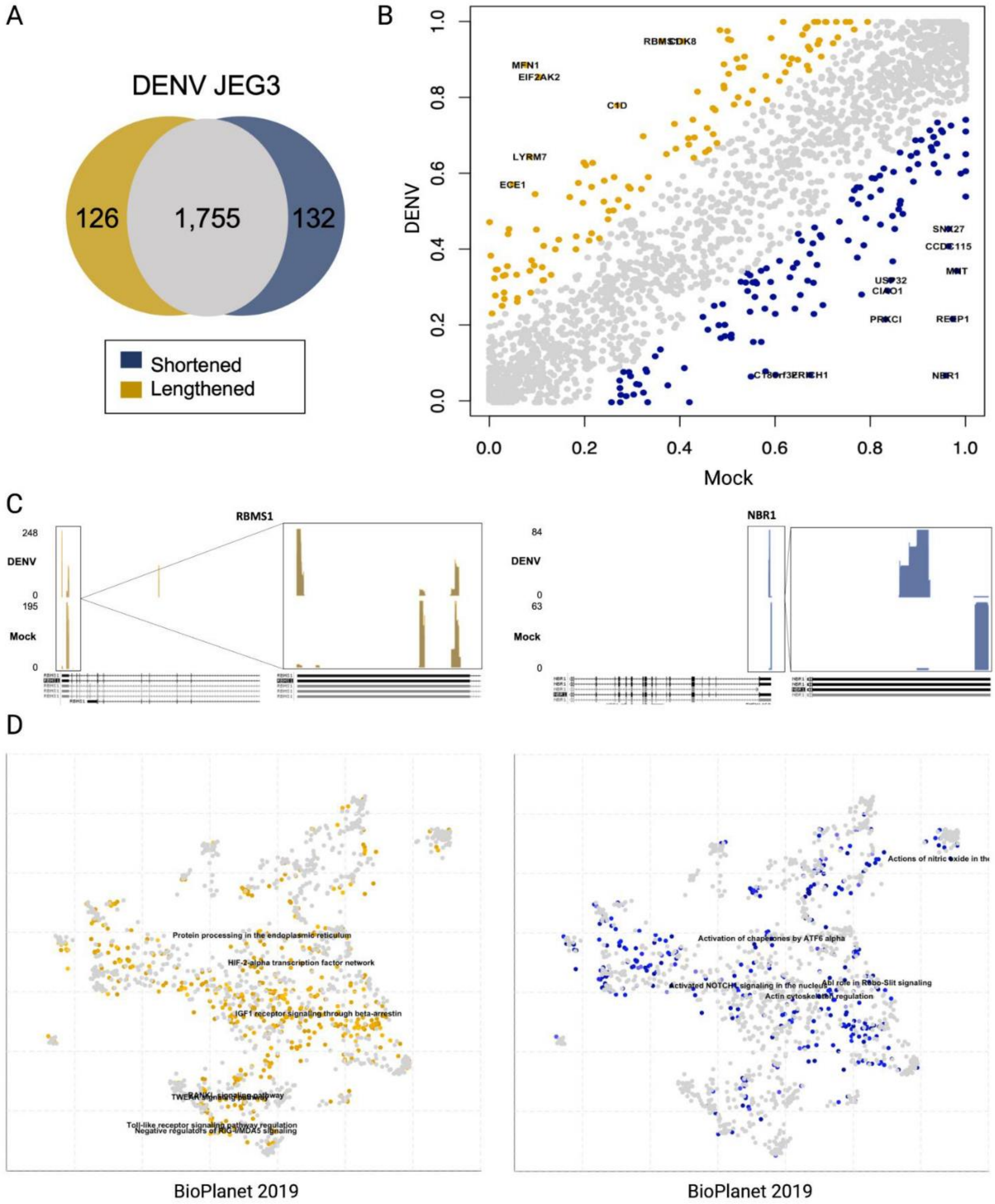

Figure S2. Alternative poly-adenylation (APA) in response to DENV infection in JEG3 cells. A) Comparison of the number of identified poly-A clusters (PACs) per condition that were lengthened or shortened by 20% or more to the overall number of PACs. B) Plots comparing the percent distal usage (PDU) of transcripts in the ZIKV or Mock condition for each time point. Blue–PDU decrease in ZIKV samples of 20% or more, indicating shortening of the 3′ UTR. Yellow–PDU increase in ZIKV samples of 25% or more, indicating lengthening of the 3′ UTR. Labelled transcripts have PDU changes greater than 50%. C) Bedgraph files for RBMS1 and NBR1 indicating the mapping of PACs in these genes which appear to be lengthened and shortened in response to ZIKV infection respectively. D) *Enrichr* scatterplots indicating enrichment of either shortening (blue) or lengthening (yellow) of 3′ UTRs of genes indicating that there appears to be shortening of transcripts involved in NOTCH signaling and lengthening of transcripts involved in protein processing in the ER and regulation of Toll-like receptor signaling [*38–40*].
